# Flow Cytometry in the Detection of Neonatal Sepsis

**DOI:** 10.1155/2013/763191

**Published:** 2013-02-03

**Authors:** Volker N. Umlauf, Stephan Dreschers, Thorsten W. Orlikowsky

**Affiliations:** Department of Neonatology, Children's Hospital, University Hospital of RWTH Aachen, Pauwelsstraße 30, 52074 Aachen, Germany

## Abstract

Neonatal sepsis remains a burden problem by showing minimal initial symptoms of subtle character, nonspecific manifestation, and diagnostic pitfalls. The clinical course can be fulminant and fatal if treatment is not commenced promptly. It is therefore crucial to establish early diagnosis and initiate adequate therapy. Besides clinical symptoms, the most reliable laboratory markers in establishing diagnosis is currently the combined measurement of CRP and a cytokine (IL-6 and IL-8). Due to their different kinetics, a diagnostic gap might occur and thus withholding antimicrobial therapy in clinical suspicion of infection is not acceptable. We therefore need parameters which unerringly differentiate between infants in need for antimicrobial therapy and those who are not. Flow cytometry promises to be a useful tool in this field, allowing the determination of different cellular, dissolved, and functional pathophysiological components of sepsis. Despite technical and methodical advances in flow cytometry, its use in clinical routine is still limited. Advantages and disadvantages of promising new parameters in diagnosis of sepsis performed by flow cytometry, particularly CD64, HLA-DR, and apoptosis, are reviewed here. The necessity of tests to be used as an “ideal” parameter is presented.

## 1. Introduction

Sepsis in the newborn is a common disorder affecting 1.1 to 2.7% of all newborns [1]. It is classified into early-onset form (EONS) within the first 72 hours of life and late-onset form (LONS) afterwards. Prematurity predisposes to sepsis: premature infants with a birth weight less than 1000 g (ELBW: extremely low birth weight infants) are particularly at risk with an inverse correlation between gestational age, birth weight, and sepsis [1]. Even late-preterm infants (LPI) have a fourfold higher risk of sepsis than term infants [2]. Thus, bacterial infections remain the most common cause for mortality and morbidity in early human life. Clinical symptoms are variable, minimal, and nonspecific [1, 3].

Deficiencies of both innate and adaptive immunity contribute to the impaired neonatal host defence (reviewed in [4]). A domination of naïve immune cells, functional impairments [5], and lower leukocyte subset numbers contribute further to an increased susceptibility [6], although basic functions, such as recognition and phagocytosis of bacteria, are already developed in the same proportion as in adults [7, 8]. 

In response to bacteremia, a systemic inflammation response syndrome (SIRS) occurs in preterm infants with rapid secretion mainly of proinflammatory cytokines (IL-1ß, IL-6, IL-8, and TNF-*α*), which leads to septic shock. Although there is evidence of a Th2-dominated, suppressed, and deficient immune response in preterm infants [4], the secretion of pro-inflammatory cytokines is detectable early in fetal development [9]. The developmentally inverse correlation between gestational age and cytokine production leads to higher concentrations in premature than in term infants [9–11] and causes a more severe inflammatory response compared to term infants [10]. The clinical manifestation is a cardiovascular dysfunction, an increasing imbalance between oxygen transport and supplies [12], altered mechanisms of metabolism, and thus leading to multiple organ failure and possible death. In chorioamnionitis the same pathomechanisms can already affect the fetus (FIRS: fetal inflammatory response syndrome) resulting in high mortality and impaired neurodevelopment in preterm infants [13].

In adult sepsis, a second phase of an anti-inflammatory reaction is a compensatory antagonistic response syndrome (CARS) associated with immunodeficiency, reduced HLA-DR expression, and increased production of anti-inflammatory cytokines [14]. In contrast to signs of immunosuppression in the blood compartments, the organ systems often were found in a hyperinflammatory state [15], which complicates to clarify the pathogenetic role of cytokines (and thus their selective modulation or blockade). In preterm infants, the CARS is weak with decreased IL-10 response and reduced cytotoxicity [10, 16]. A high percentage of adults' monocytes perishes after bacterial infection due to phagocytosis-induced cell death by apoptosis and loses the function for cytokine production [17, 18]. Neonatal monocytes are affected much less by this process, and an extended production of pro-inflammatory cytokines occurs [19], a phenomenon which is referred to as “sustained inflammation” [20].

## 2. Current State and Limitations of Diagnostics

With a delayed start of antimicrobial treatment, the fulminant course of sepsis may lead to major sequelae. Therefore, the demand for laboratory markers in the detection of sepsis is high.

The combined measurement of a cytokine (IL-6 or IL-8) and CRP is currently considered as the most reliable method with the highest sensitivity and specificity for early diagnosis of both EONS and LONS [27]. IL-6 detects sepsis at an early stage of infection with a maximum of serum levels as early as 1-2 hours after inflammation reaction has started, potentially even prior to onset of clinical symptoms [28]. The diagnostic window is short, due to the instability of IL-6, resulting in a short half life time of <20 minutes [29]. In contrast, CRP has its optimum sensitivity and specificity during the window of 24–48 hours after onset of symptoms. Thus, a diagnostic gap of several hours might occur. If CRP remains negative in repeating determinations over a period of 2-3 days, it allows early cessation of antimicrobial therapy in clinically healthy infants. Nevertheless, an early marker to identify and differentiate infants who really need treatment from those who do not would be desirable. Furthermore, current standard parameters are not adequately appropriable for prognosis and outcome. Parameters, such as monocyte HLA-DR expression and natural killer cells count, seem to be early predictive markers for the prognosis in LONS [30] or adult sepsis [31], respectively, but are under further investigation, and thus, not in clinical use.

To avoid organ complications, it is the intention of a “preventive neonatology” to detect infections at an early stage. Function-specific biomarkers, which are determined by automated flow cytometry, could therefore improve diagnosis in the future by reflecting events on a cellular level.

## 3. The “Ideal” Technology

Despite diagnostic improvements in neonatal sepsis over the past decades, flow cytometry remains a confidential diagnostic tool. Advanced flow cytometry is undeniably the best tool for analyzing signaling processes, proliferation and differentiation, cell-cell interactions, surface markers, intracellular molecules, and proteins secreted by cells. 

Modern flow cytometers are available from less than 50,000$ and have attained a high level of user friendliness. Apparatuses are smaller in size than ever, even allowing bedside (POC: “point-of-care”) measurements. 

Nevertheless, clinical situations in pediatrics or neonatology, in which flow cytometry may be used on a routine basis, are still limited. There are several reasons for it. 

Flow cytometry still suffers from its lack of standardization protocols to produce valid and reproducible data, which are mandatory for multicentric clinical studies. So, and as opposed to clinical chemistry analyzers, comparing flow cytometric studies often is impossible, since different homemade protocols (definition of a staining and gating strategy, a lysis system and an appropriate acquisition protocol) have been used [32]. Furthermore, flow cytometry still is noticed as a device mostly used by specialists in the field of immunology and hematology, and the continuously expanding clusters of differentiation rather expect too much of a clinician, working in a neonatal intensive care unit (NICU), worsened by the fact that access to flow cytometry on a 24/7 basis often is not easy in an NICU. 

## 4. The “Ideal” Parameter

Using predictive marker in diagnosis of sepsis follows two main strategies: starting an antibacterial therapy as early as possible in a case of suspected sepsis and initiating therapy only in infected patients. Thus, a high sensitivity and negative predictive value of approximate 100%, and a good specificity and positive predictive value mounted 85% are recommended [33]. In addition, standardized cut-off values are crucial, making results comparable between laboratories. 

A potential ideal marker for neonatology must exist against preanalytical limitations: due to the low weight of preterm infants with their extremely low blood volume, the test must able to work with minimal amount of blood. Experience has shown that reasonably and practicably blood volumes are at around 100 *μ*L, appropriate 2–5 drops of blood. Since the sampling is often difficult (e.g., capillary, open drop by drop), a test must have low sensitivity to the technique of sample collection and thus must be biochemically stable. Ideally, serum or EDTA blood should be used so that further routine laboratory tests can be performed from the same sample. For quick decision to treat or not to treat a patient, turnaround time needs to be kept to a minimum. Further requirement to an ideal marker contribute to the ability to differentiate between viral, bacterial, and fungal infection, and monitor the course of sepsis and duration of antibiotic treatment. In addition, the parameter should predict outcome and prognosis of infected newborns. Identify children who are susceptible to sepsis even prior to clinical symptoms would be advantageous.

The technical process of measurement needs to be performed easily (most suitable fully automated) in a routine laboratory or even by point-of-care at 24/7 availability, by nonspecial trained staff, with a minimum of preparation and working time, and at low costs. 

## 5. Cell Surface Markers

A number of cell surface markers have been studied and seem eligible for diagnostic use: a Pubmed search was performed with the keywords “neonatal sepsis” + “CD64”, or “CD11b”, or “HLA-DR”; studies since 2002 were accepted. Most studies show weak or even a lack of differentiation of gestational age, birth weight, and/or onset of sepsis, respectively, and thus, were excluded. 

In the end, we included 5 of 16 studies for reviewing and estimating sensitivity, specificity, positive, and negative predictive values of CD64 and 4/5 for CD11b (Table 1). 

### 5.1. CD64

CD64, known as Fc-gamma receptor 1 (Fc*γ*RI), binds monomeric IgG-type antibodies with high affinity [34] in the process of phagocytosis and intracellular killing of opsonized microbes. Upregulation of neutrophil CD64 is considered to be a very early step of the host's immune response to bacterial infection, increasing approximately one hour after invasion [35]. Expression of CD64 was found enhanced in neonates with blood culture proven sepsis [36], as well as clinical sepsis [17]. Sensitivity, specificity and predictive values are listed in Table 1. CD64 shows slightly better sensitivities than CRP, or IL6, both for the EONS and the LONS. Nevertheless, these differences are not convincing enough to consider CD64 the sole new sepsis marker. Combining CD64 with CRP and IL-6 increased the sensitivity and negative predictive value to 100% in LONS [18].

CD64 not only distinguished infected (median expression neutrophil CD64: 5.9) from noninfected neonates (median expression nCD64: 3.2), but also identified among the infected children culture negative sick neonates (median expression nCD64: 4.8) from those with a positive culture (median expression nCD64: 6.6) [37]. Furthermore, CD64 helped distinguishing infection from flares in autoimmune inflammatory diseases and had more limited utility for differentiating bacterial from viral infection [38]. 

CD64 is quantified as mean equivalent soluble fluorescence units and expressed as standardized CD64 index. It can be easily measured by flow cytometry in a whole-blood lysis no-wash approach, and in addition, without modifying any of the intrinsic parameters of the cells [50]. Turnaround time is a minimum of 1 hour up to 2 hours; required blood volume is merely 50 *μ*L of EDTA blood.

Standardized protocols for automated analysis and comparable cut-off values need to be established, then CD64 has the potential to become a routine parameter in diagnosing neonatal sepsis in combination with CRP and IL-6.

### 5.2. CD11b

CD11b, also known as Integrin alpha M (ITGAM) or complement receptor 3 A (CR3A), mediates inflammation by regulating leukocyte adhesion and migration. During infection, CD11b expression enhances on neutrophils and monocytes. It is implicated in phagocytosis, cell-mediated cytotoxicity, chemotaxis [51], and the complement system through its ability to bind inactivated complement component 3b (C3b) [52]. CD11b is reported to be a highly effective marker in diagnosis of early-onset neonatal infection [23, 39] with elevated expression when compared to noninfected neonates. Turnaround time of CD11b takes about 2 hours; a blood volume of less as 100 *μ*L is sufficient. Nevertheless, due to wide spreading sensitivity and specificity (Table 1), and impact on other conditions like respiratory distress syndrome [53], the role of CD11b in neonatal infections remains controversial. Highest conclusiveness might be achieved in combination with further parameters, too.

### 5.3. HLA-DR

HLA-DR is an MHC class II cell surface receptor, constituting a ligand for the T-cell receptor of T-helper cells. Monocytes strongly express HLA-DR and it is upregulated in response to signaling. Ng et al. did not find significant differences in monocyte HLA-DR expression between infected and non-infected neonates and controls [54]. In contrast, a decreased cell-surface expression of HLA-DR has been observed on circulating monocytes from neonates with sepsis by Genel et al. [30]: The percentage of monocytes expressing HLA-DR was lower in neonates with late onset sepsis and even lower in those neonates who did not survive sepsis. Patients with a monocyte HLA-DR expression <30% had a 30-fold higher risk of mortality. Establishing “normal ranges” for HLA-DR from neonates up to an age of 14 days yields increasing HLA-DR expression at the age of 2 weeks of life, possibly reflecting a maturation of these surface molecules with age [55]. Besides all, optimal results can be achieved only by relatively complex preanalytics (such as transport of example on ice, small window-of-time for measurement). Thus, HLA-DR seems to be more an early predictive prognostic marker for neonatal sepsis rather than a diagnostic marker, due to its rapid decrease issued by storage temperature and staining delay [56].

## 6. Cell Function

*Apoptosis in sepsis *is tightly regulated, and many pathogens induce the apoptosis of the host cell after the phagocytosis—a phenomenon called phagocytosis-induced cell death (PICD). On the one hand, apoptosis leads to a downregulation of the inflammatory response to sepsis due to host cell death; on the other hand, the loss of immune cells increases susceptibility to infection due to decreased ability to eliminate pathogens [57]. Higher plasma death ligands (TNF-*α*, Fas), main mediators of apoptosis pathway, associated with lower antiapoptotic molecules (Bcl-2, NF-*κ*B), are associated with increased apoptosis in neonatal sepsis [58]. Furthermore, clearance of apoptotic neutrophils through monocytes is diminished, resulting in impaired anti-inflammatory capacitiy [19]. Several *in vitro* studies reveal inhibiting or preventing apoptosis as a therapeutic option for adult sepsis (reviewed in [57]). So far, missing data for neonatal sepsis as well as the lack of automation and the costs involved in manual immunoassays make its use in general clinical practice not acceptable. Nevertheless, the apoptotic pathways offer multiple possibilities for diagnostic approaches as well as therapeutic strategies in future. 

*Toll-like receptors*, pattern recognition receptors of innate immunity, are suggested to play a major role in (adult) sepsis [59]. The data for neonatal sepsis are rare, but promising. A low basal expression of TLR2 might be associated with the particular susceptibility of neonates for infections with gram-positive pathogens. After infection, TLR2 on monocytes/phagocytes increase already at initial presentation of clinical symptoms, showed a constant high expression on monocytes in the course of neonatal sepsis, and were down-regulated after successful treatment [60]. TLR antibody staining and determination is quite simple with a short turnaround of few hours, but still not automated. Due to TLR's central position and role in sepsis pathogenesis, further studies are recommended, and might reveal promising results.

## 7. **Conclusion**

Flow cytometry allows easy measurement of a various number of parameters in the pathway of neonatal sepsis. This includes kinase assays as powerful tools to assess signal transduction and functional capabilities at the single-cell level, high-throughput screening on a multiparameter platform, multidimensional assessment of cell signaling networks to develop a mechanistic understanding of cell function, and biochemical access to rare cells [61]. Most are appropriate for understanding the pathomechanisms, less are suitable for diagnostic approach (Table 2). Currently manual immunoassays are used for determination of cell surface markers; automation is highly desirable to implement the methods in routine clinical laboratory. 

Nevertheless, none of the parameters presented is able to be *the* one parameter in diagnosis of early neonatal sepsis. So far, the combination of CRP and IL-6 remains the diagnostic resource of choice in detection of EONS and LONS. Solely CD64 seems to have the potential to complement the existing combination of CRP and a cytokine (IL-6 or IL-8) to increase sensitivity up to 100%. Larger trials to define standard measurement protocols and reference values are highly desirable. 

## Figures and Tables

**Table 1:** EONS: early-onset neonatal sepsis; LONS: late-onset neonatal sepsis; VLBW: very low birth weight neonates; PPV: positive predictive value; NPV: negative predictive value; wga: weeks of gestational age.

		Sensitivity	Specificity	PPV	NPV	Patients' characteristics/comments	Reference
EONS	CD64	96	81	71	97	*n* = 453, term	[21]
		100	86		100	*n* = 749, VLBW, LBW, term	[22]
	CD11b	100	100			*n* = 39, 29–40 wga	[23]
	CRP	81	56	48	85	*n* = 453, term	[21]
	CRP + CD64	97	71	63	98		[21]

LONS	CD64	95	88	80	97	*n* = 127, VLBW	[18]
		75			96	*n* = 749, VLBW, LBW, term	[22]
		91	83	83	91	*n* = 37; >30 wga	[17]
		70	62	59	73	*n* = 163, preterm	[24]
	CD11b	70	72	50	86	*n* = 127, VLBW	[18]
		75	100	100	86	*n* = 65, 27–38 wga	[25]
		86	100	100	68	*n* = 51, term	[26]
	IL-6	78	92	81	91	*n* = 127, VLBW	[18]
	CRP	9	83	33	50	*n* = 37; >30 wga	[17]
		65	99	96	87	*n* = 127, VLBW	[18]
	CRP + CD64	100	66			*n* = 37; >30 wga	[17]
		100	80	90	100	*n* = 127, VLBW	[18]
	CRP + CD11b	99			99	*n* = 65, 27–38 wga	[25]

**Table 2:** Cell surface markers, chemokines, cytokines, and adhesion molecules were found to play a role in neonatal sepsis pathogenesis; *might even be relevant in diagnostic use, and thus, is described in the text.

Cell surface markers		
Neutrophils	CD11b *, CD64 *	[22, 37, 39]
Lymphocytes	CD3, CD19, CD25, CD45RA, CD45RO, CD62, CD69	[6, 39–41]
Monocytes	HLA-DR *	[5, 39, 42]

Chemokines, cytokines, adhesion molecules		

	IL-1*β*, IL-1ra, IL-2, sIL-2R, IL-4, IL-5, IL-6 *, *IL-8*, *IL-10*, *TNF-*α**, 11sTNFR-p55, 12sTNFR-p75, IFNc	[43–49]
	E-selectin, L-selectin	
	Soluble intracellular adhesion moleucule-1 (sICAM-1)	
	Vascular cell adhesion molecule-1 (VCAM-1)	
